# Outpatient Therapists’ Perspectives on Working With Persons Who Are Sexually Interested in Minors

**DOI:** 10.1007/s10508-022-02377-6

**Published:** 2022-08-08

**Authors:** Alexander F. Schmidt, Susanna Niehaus

**Affiliations:** 1 Department of Psychology, Social and Legal Psychology, Johannes Gutenberg-University Mainz, Binger Str. 14-16, 55122 Mainz, Germany; 2 Department of Social Work, Lucerne University of Applied Sciences and Arts, Lucerne, Switzerland

**Keywords:** Pedohebephilia, Minor-attracted persons, Psychotherapists, Stigmatization, Treatment willingness

## Abstract

Minor-attracted persons (MAPs; i.e., people who are sexually interested in children and adolescents below the age threshold of legal consent for sexual activity) exhibit high psychological distress but report difficulties finding therapeutic help and are reluctant to start treatment due to fears of therapist stigmatization. This research sought to elucidate the link between outpatient therapists’ stigmatizing attitudes toward non-offending versus offending MAPs and therapists’ willingness to treat MAPs as well as how stigmatization was related to treatment-relevant aspects such as perceived MAP treatment needs, treatment barriers, and specific MAP treatment skills. Results from a brief, anonymous online survey conducted among *N* = 427 Swiss outpatient therapists working in the primary healthcare system are reported. Although therapists were less stigmatizing than the general public, considerable individual differences in the stigmatization of non-offending MAPs emerged. Stigmatizing attitudes toward non-offending MAPs and a perceived lack of specific treatment competences were negatively related to therapists’ willingness to treat MAPs. A network analysis revealed direct links between subjectively perceived MAP treatment competence and treatment willingness and between treatment willingness and social distance attitudes. Other stigmatizing attitudes were only indirectly linked to treatment willingness through preferred social distance. It is a paradox that therapists believe that MAPs should greatly benefit from secondary prevention but many are unwilling to provide therapy (45% in case of non-offending MAPs vs. 63% in case of offending MAPs) or do not feel competent to provide MAPs with professional help (47% with and 88% of therapists without previous MAP treatment experience). Implications for increasing therapists’ treatment willingness are discussed.

**Supplementary Information:**

The online version contains supplementary material available at 10.1007/s10508-022-02377-6.

## Introduction

Child sexual abuse is a vast social problem (Barth et al., 2013) with severe negative health-related and other consequences for victims (Manioglio, 2009). Psychotherapeutic treatment of perpetrators is considered one of the first-line tertiary prevention measures to reduce further sexual victimization of children (Gannon et al., 2019). In recent years, secondary prevention of child sexual abuse among community citizens who identify themselves as an at-risk population due to their sexual interests in minors has been coming into the focus of crime-preventative efforts (Knack et al., 2019). Yet, convincing empirical evidence for the effectiveness of preventing crime by treating individuals who feel distressed from their sexual interests in children and adolescents is still missing (Mokros & Banse, 2019). The principal idea of offering therapy for individuals suffering from psychological impairment, however, is hindered by the fact that psychological problems and disorders are negatively viewed by members of the public (Hinshaw & Stier, 2008) and—in the case of sexual interest in minors–even by mental health professionals themselves (Jahnke, 2018a). Research on the stigmatization of psychological problems has revealed that individuals who suffer from these conditions also suffer detrimental effects of stigmatization, including limited social participation (i.e., access to housing, job opportunities, or health care; Hatzenbuehler et al., 2013) as well as fostered internalization of negative stereotypes (Livingston & Boyd, 2010). Mental health-related stigma range among the top four reported barriers for help-seeking (Clement et al., 2015). This is particularly severe for persons with sexual interests in children and adolescents below the legal age of consent [in the following referred to as minor-attracted persons (MAPs)]. It has been consistently shown that sexual interest in children is a societally highly despised psychological condition (for recent overviews see: Jahnke, 2018a; Lawrence & Willis, 2021) that is associated with increased psychological distress and stigmatization stress in MAPs (e.g., Jahnke et al., 2015a, 2015b, 2015c). Furthermore, experiencing high stigmatization stress negatively impacts MAPs’ help-seeking behavior (e.g., Grady et al., 2019).

Although recently an upsurge in research on the stigmatization of MAPs living in the community has been sparked, there is a dearth of research on healthcare providers’ stigmatization of MAPs outside of secondary prevention projects or forensic institutions where individuals who have been convicted of sexual offenses against children are treated by specialists. The few existing studies primarily focus on North-American contexts. Hence, the present research sought to elucidate Swiss mental health professionals’ perspectives on and experiences with treating MAPs^1^ when working with outpatient community clients in routine healthcare therapy provision. By routine healthcare therapy provision, we refer to mental health professionals (in the Swiss health care system these are usually registered psychiatrists and psychologists) who are working with voluntary clients seeking ambulatory or outpatient treatment in non-forensic settings—a service that routinely is covered by citizen’s standard health insurances. Specifically, we were interested in the link between stigmatizing attitudes toward MAPs and outpatient therapists’ willingness to treat MAPs, in how stigmatization was related to other treatment-relevant aspects such as therapists’ perceptions of MAPs’ need for treatment and barriers to treatment, as well as specific skills for working with MAPs. To this end, we conducted a brief online survey among Swiss outpatient therapists.

### Minor-Attracted Persons Living in the Community

In terms of their sexual interests, MAPs living in the community constitute a heterogeneous group. They may exhibit sexual interest in prepubescent and/or pubescent children who are still under the age of legal consent (i.e., pedophilia, hebephilia, or pedohebephilia). The term MAP does not carry any information about the sexual offending status of an individual. The distinction between non-offending and sexually offending MAPs is separate from their sexual interest in minors. Exact prevalence estimates for MAPs in community samples are difficult to establish due to heterogeneity of sexual interests. Prevalences of attraction to minors among the male population vary as a function of their operationalization and other methodological differences between studies (for a recent overview, see Savoie et al., 2021 reporting a range between 2 and 24% across 30 studies). Generally, it has been shown that sexual interest in children (legally dependent on the age of consent within a given jurisdiction) is statistically unusual (Joyal et al., 2015) although not uncommon among the adult male population where two large-scale studies with samples of > 5000 community men yielded prevalence estimates for sexual fantasies involving prepubescent or pubescent children of 4.1% (Dombert et al., 2016) and 14.7% (Bartová et al., 2021), respectively.

#### Public Stigmatization of Minor-Attracted Persons

The term stigma originates from the Greek expression for “*mark*” and refers to negative attributes that are discrediting, eliciting avoidance of or aggression toward the carrier of the attribute who is perceived as bad, dangerous, or weak (Goffman, 1963). Stigmatization is the process of being denied social acceptance because of the discrediting attribute. This is exemplified in findings from community citizens concerning their preferred social distance from *non-offending* persons with pedophilic interests. Among a German sample, two-thirds would refrain from talking to such persons, 39% indicated they should be incarcerated, and 14% believed they would be better off dead; Among an online sample from the US, responses were more than twice as high (85%, 39%, and 26%, respectively; Jahnke et al., 2015a). Compared with other highly stigmatized psychological conditions, sexual interests in children—even among non-offending individuals—are among the most socially despised psychological problems (e.g., Boysen et al., 2020; Jahnke et al., 2015a) even when controlling for the statistical unusualness of paraphilic interests or psychological conditions that are linked to sexual offending (Lehmann et al., 2021). Empirically, although cross-sectional links between sexual interest in children and committing sexual offenses against minors have been shown in community samples (Klein et al., 2015),^2^ not every person convicted of child sexual abuse is classified as pedophilic (25–57% depending on sample, sexual interest measure, and chosen cutoff for determining pedophilia; Schmidt et al., 2013; Seto, 2018) and not every community adult with pedophilic interest commits child sexual abuse (Dombert et al., 2016; Joyal & Carpentier, 2021). Nevertheless, a widespread belief exists that strongly conflates sexual interest in children and sexual offending against minors (e.g., Jahnke et al., 2015a; Lehmann et al., 2021).

#### Perceived Stigmatization and Treatment Needs of Minor-Attracted Persons

Hitherto, all of the 14 studies reviewed by Lawrence and Willis (2021) on the stigmatization of MAPs have identified cross-sectional links to psychological impacts such as, for example, depression, anger, anxiety, despair, shame, grief, guilt, loneliness, isolation, low self-esteem, or suicidality. For instance, Jahnke et al. (2015c) reported that general psychological distress, fear of negative evaluations, self-esteem, emotional coping problems, and loneliness in MAPs were higher than in general community comparison samples. Adding to this symptomatic distress and impaired mental well-being, internalized stigma has also been associated with decreased well-being and perceived social support among MAPs (Elchuk et al., 2022; Lievesley et al., 2020; McPhail & Stephens, 2020). Strikingly, MAPs’ own experience of stigmatization distress (while actually overestimating the public stigma) has been cross-sectionally linked to increased risk factors for committing child sexual abuse such as, for example, loneliness, emotion regulation problems, and low self-esteem (Jahnke et al., 2015a).

Despite the expressed need for therapeutic help from MAPs (e.g., 12% of the community respondents in Dombert et al., 2016 who indicated sexual interest in prepubescent children had thought about seeking treatment due to their sexual interests), MAPs were reluctant to disclose their sexual interest to health professionals (e.g., only about half of the participants from Jahnke et al., 2015c were willing to disclose their sexual interest to a therapist) and, more importantly, reported substantial difficulties in finding adequate professional help (Goodier & Lievesley, 2018). For example, 75% of the MAPs surveyed by Levenson and Grady (2019a) indicated that they had sought professional help but less than half of them found their treatment to be helpful. Resembling findings for general psychotherapy patients, characteristics of treatment that MAPs experienced as helpful were described as nonjudgmental therapists, being viewed holistically in a person-centered way, therapists’ adequate knowledge about minor attraction, certainty about confidentiality, and manageable financial constraints (Levenson & Grady, 2019a). Although controlling, and also understanding or reducing attraction to minors were common treatment goals particularly on the side of the professionals, MAPs prioritized general mental health issues such as depression, anxiety, loneliness, low self-esteem, and (sexual) self-regulation as well as coping with stigmatization stress (B4U-ACT, 2011; Grady et al., 2019). Relatedly, disclosure of sexual interests was only perceived as conducive to reducing personal distress if MAPs felt it was followed by adequate social support (Elchuk et al., 2022).

#### Therapists’ Stigmatization of Minor-Attracted Persons

Only few studies so far have explored therapeutic professionals’ perceptions of MAPs and their treatment needs. Published studies have largely focused on professionals working in specific sexual abuse prevention settings (Beggs Christofferson, 2019; Goodier & Lievesley, 2018; Levenson & Grady, 2019b; Parr & Pearson, 2019) or students about to graduate as social workers or counselors (Walker et al, 2022). Three studies by Lievesley et al. (2022), Stephens et al. (2021), and Roche and Stephens (2022) that took approaches most similar to that in the present study have focused on varying healthcare practitioner groups, however, consisting of sizeable subsamples of practitioners not (yet) specialized for psychotherapeutic treatment. In Lievesley et al., (2022; *N* = 220) roughly half of the sample (46%) classified themselves as primary medical healthcare specialists (e.g., physicians), and 19% of the participants in Roche and Stephens (2022; *N* = 101) were students, social workers (24%), or psychologists or psychotherapists (roughly 50%). The hitherto largest mental health clinician sample so far (Stephens et al., 2021; *N* = 309) consisted of roughly a third of practitioners who identified as social workers or professionals other than registered clinicians, psychologists, or psychiatrists, who made up the remaining sample. However, this experimental study focused primarily on mandatory reporting decisions and not on willingness to treat.

Results from these broader healthcare practitioner studies revealed that MAPs’ abovementioned skepticism concerning therapists’ willingness to treat them and MAPs’ fear of being stigmatized or (unnecessarily) reported to legal authorities may not be unfounded. Mental health professionals and students in training indicated to being willing to report MAPs to legal authorities due to explicit stigmatization and/or a lack of knowledge about the administrative framework concerning reporting standards (e.g., Beggs Christofferson, 2019; Stephens et al., 2021; Walker et al., 2022). For example, clinicians’ decisions to officially report a client who disclosed sexual interest in children were a function of the number of client risk factors (i.e., child sexual exploitation material use, access to children), although, even in the absence of any risk factor, 12% of the clinicians indicated that they would report their client (Stephens et al., 2021). In a study by Beggs Christofferson (2019), 14% of the surveyed therapists considered reporting a client who disclosed sexual interest in children, even if this meant to break the relevant confidentiality law. Among social service students, 54% agreed to report “a pedophile” client (no sexual offense was mentioned) to the police (strikingly, this rate was reduced to 7% when the case in question was labeled as someone with sexual interest in children but who never has committed any offense against children; Walker et al., 2022).

In terms of therapist competency, only between roughly a quarter and 43% of practitioners answered correctly that pedophilia is a sexual attraction to children below the age of 11 and such general knowledge deficiencies about aspects related to minor attraction were associated with stigmatizing attitudes (Lievesley et al., 2022). Therapists’ stigmatization of MAPs, in turn, was negatively related to their willingness to accept MAPs for treatment (Roche & Stephens, 2022). Further evidence for considerable reluctance to work with MAPs was reported by Stiels-Glenn (2010) who found that 95% of psychotherapists they surveyed refused to work with “pedophiles”. Jahnke et al., (2015b) showed that 20% of psychotherapists in training were not willing to treat someone with sexual interest in children who has never committed child sexual abuse, and 60% were unwilling to treat someone who has. Although preliminary experimental work showed that lacking accurate knowledge about MAPs and some stigmatizing attitudes were amenable to simple interventions among clinicians (Jahnke et al., 2015b; Levenson & Grady, 2019b), such efforts were not always successful (Walker et al., 2022). Therapists’ perceived competence was associated with willingness to treat MAPs on a zero-order level but had to be removed due to collinearity in a multivariate model controlling for stigmatizing attitudes. In this multivariate model, it was revealed that treatment willingness was statistically linked to therapists’ anticipated comfort and therapists’ regard of mental health issues as an important treatment focus, whereas focally controlling sexual attractions was negatively related to therapists’ willingness to treat MAPs (Lievesley et al., 2022).

### Current Study

In the literature, stigmatization of MAPs becomes apparent as a crucial variable likely to be detrimental to MAPs’ treatment seeking behavior on the one hand, and healthcare professionals’ willingness and competence to deliver treatment on the other hand. To complement the current literature, we sought to draw a large sample exclusively consisting of mental health specialists (i.e., psychologists, psychiatrists) who have been working with outpatient clients outside of specialized forensic treatment settings. Thereby, we sought to explore the provision of routine psychotherapeutic treatment by regularly trained therapists (i.e., not specialized for treating MAPs) in a European healthcare system with less stringent reporting laws than in English-speaking contexts (on which the literature has so far mainly focused).^3^ In this way, we aimed to describe the attitudes, expectations, and experiences of a population who, in principle, should be most sympathetic and accustomed to people with mental health problems because dealing with psychological distress is an inherent part of their job profile and plays a central role in their professional training. This notion is corroborated by higher stigmatization of MAPs among general medical practitioners than mental health specialists (Lievesley et al., 2022). Notably, prior studies have included less stringently selected samples of healthcare practitioners and as a result included a much more heterogeneous and potentially more stigmatizing group of healthcare-related occupations and—in cases in which multivariate analyses have been conducted—focused on only a few, select dependent variables in multiple regression analyses.

Based on prior findings, we hypothesized that (a) stigmatizing attitudes toward MAPs as well as (b) a lack of specific treatment competencies and treatment experience with MAPs would be negatively related to therapists’ willingness to therapeutically treat MAPs and MAP treatment-related attitudes and expectations. Moreover, we expected that (c) therapists would be less willing to treat sexually offending as opposed to non-offending MAPs and (d) therapists would exhibit less stigmatizing attitudes than the general public (as shown by comparison with German community data from Jahnke et al., 2015a). We sought to (e) further gauge Swiss outpatient therapists’ stigmatization of MAPs against a small sample of Russian sex therapists who had been tested on the same measures (Koops et al, 2016) to be able to position the Swiss therapists among lay citizen and specialized therapeutic comparison groups. Finally, as the complex interplay of therapists’ perspectives on and working experiences with MAPs has not yet been examined from a comprehensive multivariate perspective, we aimed (f) to run an exploratory network analysis (Borsboom et al., 2021) on our main variables. By exploring the network of interrelations between the surveyed attitudes, expectations, and experiences, we sought to identify possible intervention targets in therapist training and further education that may be conducive to both increasing primary healthcare professionals’ willingness to work with the underserved population of MAPs (McPhail et al., 2018) and reducing therapists’ stigmatization of MAPs (Jahnke, 2018a).

## Method

### Participants

Swiss outpatient therapists were recruited via member lists or mailings from relevant psychological, sexological, and medical professional associations in Switzerland [Federation of Swiss Psychologists (FSP), Association of Swiss Psychotherapists (ASP), Société Suisse de Sexologie (SSS), Swiss Society of Child and Adolescent Psychiatrists (SGKJPP) and 19 additional cantonal psychiatric associations as the Swiss psychiatric umbrella organization at the federal level opted not to send the survey link to their members]. Eligible participants were psychological, psychiatric, or sexological therapists who had worked or were currently working with community outpatients. Links to the survey were provided to potential participants via email. They were informed that the anonymous online survey lasting 10–15 min tapped into therapists’ experiences and challenges when working with MAPs. Survey instructions also explicitly addressed therapists without therapeutic experience with MAPs. The survey was online from June 2019 to October 2020. Participation was voluntary, and no compensation was offered.

Based on 6295 emails that had been sent out, 815 (12.9%) participants clicked on the link, 641 (10.2%) started to fill in the questionnaire, and 438 (6.9%; 53.7% of those who had followed the invitation link) completed the whole survey. Notably, the response rate likely represents an underestimation of the eligible population because not every member was actually reached by email due to outdated contact information. Additionally, some email recipients might have been members of several of the professional associations at the same time, thus receiving duplicate emails, and some of the contacted medical associations did not distinguish between psychiatric and other medical subdivisions so that an unknown number of non-eligible medical professionals (those not working in psychiatric/psychotherapeutic contexts) may also have been contacted.

From all participants who finished the survey, eight were deleted as they had never worked with outpatients. Three additional respondents who chose the non-binary sex option were excluded as their small number precluded any statistical analyses. The effective sample, thus, consisted of *N* = 427 Swiss therapists who had worked with outpatients from the community during their professional career.

On average, participants were 53.9 (*SD* = 10.9) years of age, ranging between 31 and 87 years with men’s mean age being higher than women’s (Table 1). They had been working as a therapist for 3 to 40 years (*M* = 22.8; *SD* = 10.1). Male therapists indicated more therapy experience than did female therapists. Roughly two-thirds of the participants were psychological therapists (63%), one-third medical therapists (34.2%) and only a small minority of therapists from other disciplines (e.g., sexologists; 2.8%). Men reported more frequently being medical professionals whereas women were more frequently psychologists. The majority worked exclusively with adults and only roughly a quarter worked with children and juveniles (26.3%). Two-thirds of the sample worked in independent practice as opposed to being employed by another group or organization. More than half of the therapists reported working in major Swiss urban areas and the large majority was situated in Swiss German language regions (75%; see Table 1 for more details).

**Table 1:** Overview of descriptive therapist data and sex differences

	Total *N* = 427		Female therapists *n* = 293		Male therapists *n* = 134		Statistical difference *U*/χ^2^	
	*M*/*n*	*SD*/%	*M*/*n*	*SD*/%	*M*/*n*	*SD*/%	*p*	*r*^c^/*V*
Age (years)	53.9	10.9	52.7	11.0	56.5	10.3	< .001	.20
Experience (years)	22.8	10.1	21.2	9.9	26.1	9.8	< .001	.28
Profession							< .001	.21
Medicine	146	34.2	81	27.6	65	48.5		
Psychology	269	63.0	205	70.0	64	47.8		
Other	12	2.8	7	2.4	5	3.7		
Patients							.275	.08
Adult	272	63.7	181	61.8	91	67.9		
Child & youth	65	15.2	44	15.0	21	15.7		
Both	90	21.1	68	23.2	22	16.4		
Work form^a^							.038	.13
Employed (practice)	58	14.6	47	17.4	11	8.7		
Employed (institution)	80	20.2	57	21.1	23	18.3		
Independent (practice)	258	65.2	16	61.5	92	71.0		
Working city size (population)^b^							.391	.10
≤ 5000	23	5.4	17	5.8	6	4.5		
5001–10,000	27	8.7	30	10.3	7	5.3		
10,001–40,000	121	28.5	83	28.4	38	28.6		
40,001–100,000	73	17.2	51	17.5	22	16.5		
≥ 100,001	171	40.2	111	38.0	60	45.1		
Language region							.407	.07
Swiss German	319	74.7	214	73.0	105	78.4		
French	90	21.1	67	22.9	23	17.2		
Italian	18	4.2	12	4.1	6	4.5		

^a^*N* = 396;^b^*N* = 425;^c^rank biserial correlation from Mann–Whitney* U* tests

### Measures and Survey Procedure

The survey was programmed and presented via SoSciSurvey (Leiner, 2019). Items are described in the order they were presented during the survey. Because there are three different major languages spoken in Switzerland–Swiss German (*n* = 319), French (*n* = 90), and Italian (*n* = 18)–participants could choose their preferred survey language prior to starting the survey. French and Italian versions had been translated from the German version by native speakers and backtranslations had been checked by the authors (French version) and an Italian-speaking research assistant. In order to maximize participant turnout and reduce the burden on professionals’ time resources, we tried to keep the survey as brief as possible. Depending on participants’ answers the survey consisted of a maximum of 54 items. Having chosen their survey language, participants were asked to report demographic characteristics relating to their professional practice (i.e., age, sex, language region where they work, profession [medical, psychological, sexological, other], patient target group [adults, adolescents, children], current work status with outpatients, duration of past experience with in- and outpatients, population size of working town) (Table 1).

#### Items on Stigmatizing Attitudes

The demographic survey section was followed by a set of 19 items tapping into stigmatizing attitudes toward individuals with sexual interest in children. All items in the stigma section (and the following sections if not stated otherwise) had to be answered on a Likert scale from 1 (*does not apply at all*) to 7 (*does apply completely*). Participants were instructed that the following items referring to individuals with sexual interest in children related exclusively to individuals who had *never* committed any sexual offense involving children such as contact child sexual abuse or use of child sexual exploitation material (i.e., “child pornography”). Items consisted of shortened versions from scales that had been validated in prior research on stigmatizing attitudes toward MAPs and were presented in a fixed random-order.

Four items were taken from the *Social Distance Scale* (SDS; Jahnke et al., 2015a) assessing the preferred amount of social distance individuals would like to keep from persons with sexual interest in children (see Table 2 for item content; the two original items inquiring whether these persons were better off dead or to should be incarcerated were left out because they also tap into punitive attitudes which were assessed separately). The SDS has been shown to be associated with other stigmatizing attitudes toward individuals with sexual interest in children (Jahnke et al., 2015a; Jahnke, 2018b) and has consistently shown that people have preferences for larger social distances from people with pedophilia compared to various other stigmatized groups in community samples (Jahnke et al., 2015a; Lehmann et al., 2021) or controls with sexual interest in adults (Jahnke, 2018b). Internal consistency for the aggregated scale in the present study was good (McDonald’s *ω* = .87; Table 3).

**Table 2:** Descriptive item overview of (non)stigmatizing attitudes toward non-offending MAPs and comparison with German community citizens or Russian sex therapists

Item	Swiss outpatient therapists *N* = 427 (OT)				Jahnke et al., (2015a), Study 1 German community citizens^a^ (CC)					Koops et al. (2016) Russian sex therapists^b^ (ST)					OT vs. CC		OT vs. ST	
	*M*	SD	Uncertain (%)	Agree (%)	*M*	SD	Uncertain (%)		Agree (%)	*M*	SD	Uncertain (%)		Agree (%)	*p*	*d*	*p*	*d*
Intentionality^c^																		
Deliberate decision to have pedophilic interests	.76	1.19	4.9	4.6	2.41	2.07	15.3		30.3	2.35	1.79	23.1		23.1	< .001	− 0.91	< .001	− 1.29
Have the choice whether they have pedophilic interests or not	.93	1.27	8.4	4.0	2.41	2.08	16.7		29.4	3.08	1.60	19.2		46.2	< .001	− .80	< .001	− 1.67
Something that one can choose	.62	1.06	3.0	3.1	2.21	2.07	16.0		26.2	2.23	1.77	26.9		23.1	< .001	− .89	< .001	− 1.45
Social Distance Scale^ce^																		
Accept as colleague	2.30	2.07	14.5	31.2	1.43	1.76	11.5		14.4	1.32	1.35	8.0		8.0	< .001	.47	.002	.48
Accept as friend	2.37	1.91	20.4	27.6	.96	1.43	8.4		7.1	1.20	1.53	8.0		12.0	< .001	.88	.113	.62
Accept as neighbor	2.95	1.97	18.7	40.5	1.24	1.63	11.2		10.4	1.28	1.40	8.0		8.0	< .001	.98	< .001	.86
Talk to them	4.46	1.69	11.5	75.4	2.61	2.12	16.2		34.2	2.56	1.64	16.0		32.00	< .001	.93	.001	1.13
Dangerousness^d^					n/a					n/a								
Link to child sexual abuse	4.75	1.80	15.2	58.8														
Will lead to child sexual abuse	3.06	1.56	18.3	20.1														
Have sex with children	3.25	1.61	24.4	19.4														
Punitive Attitudes^d^					n/a									n/a				
Inform citizens about child sexual offenders as neighbors	3.10	1.91	14.8	26.0														
Preventive detention	1.97	1.40	8.2	6.8														
Forbid to work w/ children	5.90	1.65	4.4	84.7														
Mandatory psychotherapy	3.91	2.02	17.8	40.3														
Openly available sex offender registry w/ name, photo, address	1.70	1.24	6.1	4.7														
Chemical castration	2.00	1.50	8.9	8.7														
Deviance^d^					n/a					n/a								
Normal w/ rare inclinations (recoded)	4.36	2.02	10.5	48.9														
Are sick	4.65	1.91	15.9	57.1														
Need treatment	5.78	1.66	10.1	80.3														

*MAPs* Minor-attracted persons; *n/a* Data not available in comparison studies

^a^*N* ≥ 841, for exact sample sizes refer to Jahnke et al., (2015a, Study 1, Table 1); ^b^*N* ≥ 25, for exact sample sizes refer to Koops et al., (2016, Table 1); ^c^items have been rescaled (Likert scales from 1–7 to 0–6) to fit the scale formats in Jahnke et al., (2015a) and Koops et al. (2016), Uncertain (score 3), Agree (scores 4–6); ^d^Likert scales from 1 to 7, Uncertain (score 4), Agree (scores 5–7); ^e^raw values (not recoded to match comparison samples)

**Table 3:** Descriptives, interorrelations (Kendall’s *τ*), and internal consistencies (McDonald’s *ω*; main diagonal in brackets)

	*M*	*SD*	1	2	3	4	5	6	7	8	9	10	11	12	13	14	15	16	17	18	19	20	21
1. Therapist sex^a^	–	–	–																				
2. Therapist age	53.9	10.9	− **.13**	–																			
3. Tx experience (years)	22.8	10.1	− **.18**	**.55**	–																		
4. Profession^b^	–	–	**.21**	− **.1**	− **.17**	–																	
5. Dangerousness	3.69	1.23	.04	.01	.03	− .05	− .59																
6. Intentionality	1.77	.98	− .03	**.13**	**.12**	.07	**.11**	− .78															
7. Deviance	4.93	1.31	**.12**	− .07	− .03	− .09	**.33**	.04	− .54														
8. Punitive attitudes	3.1	1.09	**.13**	− .07	− .03	.03	**.32**	**.20**	**.36**	− .75													
9. Social distance scale	3.98	1.61	**.15**	− **.13**	− .06	− .03	**.37**	**.13**	**.38**	**.37**	− .87												
10. MAP Tx experience^c^	–	–	− **.23**	.09	**.10**	− .07	.00	.00	− .07	− **.10**	− **.10**	–											
11. Tx competence	2.7	1.82	− **.22**	.07	.08	.02	− .08	.01	− .10	− **.11**	− **.18**	**.43**	–										
12. Tx success expectation	5.3	1.18	.07	− .09	− .06	**.13**	− .05	− **.13**	.01	− .05	− **.12**	.07	**.15**	− .85									
13. Tx willingness	3.47	2.04	− **.23**	.02	− .01	**.10**	− **.13**	− .03	− **.16**	− **.15**	− **.26**	**.36**	**.56**	**.12**	− .87								
14. Tx barriers skills & liability	4.71	1.40	**.20**	− **.12**	− .07	− .02	**.14**	− .02	**.17**	**.20**	**.27**	− **.25**	− **.43**	− .05	− **.38**	− .74							
15. Tx barriers effort	2.84	1.31	− .02	.03	.04	− .02	**.22**	**.17**	**.19**	**.26**	**.28**	00	− .06	− .07	− **.14**	**.23**	(.74)						
16. MAP problems behavioral	3.42	1.14	.08	− .02	− .01	.04	**.21**	**.18**	**.21**	**.23**	**.19**	− .06	− .04	− .08	− .07	**.11**	**.25**	− .82					
17. MAP problems intimacy	5.41	.84	.02	− .04	.02	− .04	.07	− .09	**.12**	.03	.03	.09	.07	**.16**	.05	− .01	.03	− .01	− .67				
18. MAP Problems Affective	4.76	1.04	.02	− .05	.02	.04	− .03	− .05	.05	.05	− .01	.07	.09	.06	.09	.05	.07	**.14**	**.32**	− .82			
19. MAP problems interpersonal	4.99	1.07	.01	.04	.08	− .05	**.19**	.05	**.23**	**.18**	**.18**	**.11**	**.13**	− .01	.04	− .01	**.16**	**.28**	**.35**	**.32**	− .74		
20. Tx success expectation medication	3.69	1.08	.05	− **.14**	− **.11**	.00	.07	.00	**.1**	**.16**	.08	.01	.06	.08	.03	.09	**.11**	**.23**	.01	**.17**	**.13**	− .65	
21. Secondary prevention needed	6.22	1.07	.09	− .09	− .08	.02	− .01	− **.15**	.04	.01	.01	.04	.00	**.28**	.00	**.11**	− .05	− .05	.16	.06	.04	.06	− .57

*N* = 427. *Tx* Treatment; *MAP* Minor-attracted person; correlation coefficients *τ* ≥ │.07 │ are statistically significant (*p* < .05); conventionally at least small effects *τ* ≥ │.10│ in bold

^a^Higher values indicate female therapists; ^b^dichotomized: 0 psychological therapists vs.1 medical therapists (*N* = 415); ^c^0 no 1 yes; all other items/scales are seven-point Likert scales (1 ‘does not apply at all’ to 7 ‘does apply completely’)

**Table 4:** Descriptive overview of therapists’ treatment experiences with MAPs

	Total *N* = 427		Female therapists *n* = 293		Male therapists *n* = 134		Statistical difference χ^2^/*U*	
	*n*/*MD*	%/*IQR*	*n*/*MD*	%/*IQR*	*n*/*MD*	%/*IQR*	*p*	ϕ/*r*/*d*
Experience w/ MAPs	178	41.7	100	34.1	78	58.2	< .001	.23
Experience male MAPs	174	40.7	96	32.8	78	58.2	< .001	.24
Experience female MAPs	27	6.3	19	6.5	8	6	.839	.01
Patients from therapists w/ MAP treatment experience^a^								
% sex. interest adults^b^	50	99	50	99	60	96.5	.566	.05
% voluntary treatment^bc^	36.5	100	31.5	100	45	100	.771	.02
% sex. interest revealed^bd^	20	100	0	50	50	100	.005	.23
% CCSA revealed^be^	5	61.5	0	50	20	73.75	.159	.12
% CSEM revealed^bf^	20	70	0	52.5	40	90	.024	.19
# male MAPs treated^gh^	2	3	2	2	2	4	.306	.16
# female MAPs treated^h^	0	0	0	0	0	0	.786	− .05

*MAPs* Minor-attracted persons; *IQR* Interquartile range; *CCSA* Contact child sexual abuse; *CSEM* Child sexual exploitation material use

^a^median and *IQR*; ^b^rank biserial correlations from Mann–Whitney *U* tests; ^c^*N* = 176; ^d^*N* = 175; ^e^*N* = 171; ^f^*N* = 172; ^g^*N* = 177 (one extreme outlier removed); ^h^Cohen’s *d* from bootstrapped independent *t*-tests based on 1000 samples

Fifteen further items were taken from the item set tapping into stigmatization against MAPs from Imhoff (2015). We used three items from each of the *Dangerousness*, *Intentionality*, and *Deviance* subscales as well as six items from the *Punitive Attitudes* subscale (see Table 2 for item content for each scale). Items were selected from the original scales based on the highest part-whole corrected item-total scale correlations (Imhoff, personal communication). The Dangerousness subscale assesses how likely sexual interest in children will lead to child sexual abuse. The Intentionality subscale taps into respondents’ beliefs that persons with sexual interest in children have freely chosen their sexual inclinations. The Deviance subscale measures how strongly individuals believe that sexual interest in children is abnormal or sick. Finally, the Punitive Attitudes subscale taps into wishes for punitive action against MAPs. These stigmatizing attitudes have been shown to be meaningfully associated with attitudes toward sexual offenders and moral disengagement from individuals with sexual interest in children (Harper, Bartels, & Hogue, 2018). Stigmatizing attitudes were particularly pronounced when the label pedophilia (vs. sexual interest in children) was used (Imhoff, 2015; Imhoff & Jahnke, 2018a, 2018b) or when MAPs were portrayed as having committed sexual offenses (vs. not; Boardman & Bartels, 2018). The internal consistencies of the four aggregated subscales were satisfactory for the Intentionality and Punitive Attitudes scales (*ωs* = .78 and .75, respectively) but not for the other two subscales (Dangerousness and Deviance with *ωs* = .59 and .54, respectively; Table 3).

#### Items on Secondary Prevention-Related Beliefs and Experiences with Treating Minor-Attracted Persons

Subsequently, a block of newly formulated items explicitly referring to persons who voluntarily seek treatment because of their sexual interest in children and adolescents (< 16 years of age) was presented. It started with two items tapping into the perceived effectiveness of secondary prevention programs for adults who had not committed any sexual offense and with sexual interest in children at least five years younger than themselves (*ω* = .85 for the combined items; Table 3). Next, three items assessed the therapeutic effectiveness of psychopharmacological treatment options; (a) sex-drive reducing medication, (b) selective serotonin reuptake inhibitors (SSRIs), and (c) antipsychotic medication (*ω* = .65 for the aggregated three items; Table 3). These items were followed by questions addressing whether participants felt competent in treating problems associated with sexual interest in minors and a dichotomous (yes/no) question on whether therapists had acquired specific treatment skills for MAPs (in case the latter item was affirmed, participants also were asked using the previously described seven-point Likert scale where they had acquired these skills from, i.e., (a) their studies, (b) their therapy education, (c) a specific training or workshop, (d) forensic practice, (e) supervision/intervision, (f) self-studying the literature, (g) conferences, (h) their general practice). This subsection closed with two questions on therapists’ willingness to treat non-offending and offending MAPs (*ω* = .87 for the combined two items; Table 3).

These items were followed by two items measuring therapists’ reported number of treated outpatient and inpatient MAP clients. If a therapist reported having treated at least one MAP, the therapist was asked to estimate the percentage of MAPs they had treated who (a) had additional sexual interests in adults, (b) had sought treatment voluntarily (without judicial orders), (c) had revealed their sexual interests in minors when starting their therapy, (d) had indicated having committed contact child sexual abuse and (e) had reported having used child sexual exploitation material. All participants were then asked (yes/no) whether they referred MAPs for whom they could not or would not like to offer treatment to a different treatment institution. If a therapist indicated having redirected a patient or patients, we used the abovementioned seven-point Likert scale to inquire where patients were referred to: (a) MAP-specific treatment options (i.e., akin to the German *Prevention Project Dunkelfeld*; Beier et al., 2021), (b) forensic programs such as forensic outpatient treatment centers, (c) psychiatric colleagues, (d) neurological colleagues, and (e) therapists specialized in treatment of sexual problems. Therapists who did not refer MAPs to other institutions were asked whether they would like to refer MAPs they cannot offer a treatment opportunity to other therapists but did not know about suitable places (yes/no). Finally, we asked two items about how helpful secondary prevention opportunities for MAPs were perceived and whether therapists thought that more of such treatment approaches were needed (*ω* = .57 for the aggregated two items; Table 4).

#### Items on Minor-Attracted Persons’ Treatment-Relevant Problem Severity

We asked therapists to rate the following 17 psychological problems that MAPs seeking treatment might exhibit in terms of their perceived problem severity: (a) difficulties in refraining from using child sexual exploitation material, (b) difficulties in abstaining from committing contact child sexual abuse, (c) problems resulting from a lack of satisfying sexual experiences that could be realized, (d) problems from keeping their sexual interests in minors secret, (e) problems with intimate relationships with adults, (f) problems resulting from having experienced child sexual abuse in their childhood/youth, (g) isolation/social withdrawal, (h) affective problems (e.g., depression), (i) anxiety (e.g., social anxiety), (j) substance abuse problems, (k) personality disorders, (l) self-control deficits/emotion regulation problems, (m) chronic criminality (antisociality), (n) sexual dysfunctions, (o) hypersexuality, (p) cognitive disabilities, and (q) problems from the psychotic spectrum. These items were planned to be subjected to an exploratory factor analysis. Finally, participants were given the option in an open-response item of freely listing what they perceived as the most important problem not mentioned in the prior list, then rating its severity if they wanted to do so.

After this item set three items followed that inquired as to whether therapists’ considered (a) involving the close relatives of their MAP patients into treatment helpful, (b) MAPs’ masturbation to legal depictions of minors (such as pictures from advertisements, manga or comic books, computer animations) problematic, and (c) MAPs’ use of sex dolls as an unproblematic way of living their sexuality.

#### Items on Perceived Treatment Barriers

The survey closed with a set of seven items tapping into personal reasons that hinder therapists from treating MAPs. Here, therapists rated whether (a) they were not qualified enough to treat this group, (b) they felt uncomfortable working with these people, (c) they were worried about treatment errors on their side that might lead to the victimization of children, (d) they feared that they could be personally held liable for any treatment error, (e) they were worried about what their other patients would think if they became aware the therapist also treated MAPs or when other patients thought that they might encounter MAPs in the practice, (f) MAPs are unpredictable, (g) MAPs are too labor-intensive. These seven items were also planned to be subjected to an exploratory factor analysis. Finally, participants could end with an open-ended question giving room for further voluntary remarks.

### Statistical Analyses

Descriptive data were cross-tabulated and *χ*^2^-tests, nonparametric Mann–Whitney *U* tests, or bootstrapped *t*-tests were used for group comparisons with *r*_ϕ_ and Cramer’s *V,* rank biserial correlations, or Cohen’s *d* as the respective effect sizes. For comparisons with data from preexisting samples we used *t*-tests and nonparametric Kendall’s *τ* coefficients for zero-order intercorrelations of study variables. Throughout the manuscript when we report descriptive answer tendencies from the Likert-scaled items or subscales, (non)agreement rates refer to answers above (below) the scale midpoint and uncertainty rates refer to the scale midpoint.

Exploratory factor analyses were run with oblique factor rotations (promax) using parallel tests and visual inspection of the scree plots (i.e., elbow criterion) to ascertain the number of factors to be extracted. Further multivariate analyses were conducted within a cross-sectional network analysis. The resulting network represents pairwise conditional associations (edges) between the study variables (nodes) controlling for the influence of all other variables in the network. Network analysis is particularly well suited for exploratory analyses of the interrelations of a set of variables with varying scale levels (Borsboom et al., 2021). We used a mixed graphical model approach (Haslbeck & Waldorp, 2020) for network estimation that allowed incorporation of a mixture of continuous and binary categorical data. Based on Isvoranu and Espkamp’s (2021) recommendations for achieving precise networks (i.e., maximize the detection of true edges) with lower sample sizes, the estimated network was based on zero-order regularized nodewise regressions with Extended Bayesian Information Criterion (EBIC) model selection and a hypertuning parameter of *γ* = .25. The network plot was generated using standard settings in JASP version 0.16.1 (JASP Team, 2022). To further quantify how well a node was directly connected to other nodes in the network structure, we investigated absolute strength as a centrality measure. Finally, we gauged the accuracy of the edge weight and centrality estimates conducting nonparametric and case-drop bootstrapping, respectively, based on each 1000 bootstrap samples.

## Results

### Exploratory Factor Analyses

Exploratory factor analysis (principal axis factoring, oblique promax rotation) with the items tapping into the severity of MAPs’ treatment-relevant problems (KMO = .84, Bartlett’s test *χ*^2^(120) = 2,41.7, *p* < .001) yielded four factors according to a parallel test and visual inspection of the scree plot. After omitting the item on difficulties in abstaining from committing contact child sexual abuse due to cross-loadings on three factors, model fit was satisfactory [RMSEA .05, CI_90%_ (.04; .07), TLI = .093]. The four resulting factors (see Electronical Supplement Table S1 for details on the factor structure) were labeled as (1) *MAP Sexual and Behavioral Mental Disorders* (consisting of the antisociality, sexual dysfunctions, hypersexuality, cognitive disabilities, psychotic spectrum items; *ω* = .82), (2) *MAP Intimacy Problems* (difficulty desisting from child sexual exploitation material use, lack of satisfying sexual experiences that can be legally realized, problems from keeping their sexual interest in minors secret, problems with intimate relationships with adults; *ω* = .67), (3) *MAP Affective Problems* (loneliness, depression, anxiety, substance abuse; *ω* = .82), and (4) *MAP Interpersonal Problems* (self-control deficits/emotion regulation problems, personality disorders, problems from having experienced child sexual abuse in their childhood/youth; *ω* = .74; see Table 3 for more descriptive data and intercorrelations of the subscales).

Conducting the same exploratory factor analysis with the seven-item set tapping into participants’ perceived treatment barriers [KMO = .71; Bartlett’s test *χ*^2^(21) = 735.1, *p* < .001] revealed a two-factor solution (see Electronical Supplement Table S2 for details on the factor structure) based on the inspection of the scree plot and a parallel analysis. The model fit fell slightly below common cutoffs for acceptable model fit [RMSEA .11, CI_90%_ (.09; .15), TLI = .084]. The two resulting factors were labeled as (1) *Treatment Barriers Skills and Liability* (consisting of the items tapping into lacking treatment qualification for MAPs, feeling uncomfortable with MAPs, worrying about treatment errors that might lead to victimization of children, and worrying about being held liable for treatment errors; ω = .74) and (2) *Treatment Barriers Effort* (unpredictable behavior of MAPs, treating MAPs is too much work, worrying about non-MAP patients thoughts’ when they become aware of MAPs being treated by their therapist; *ω* = .74; see Table 3 for more descriptive data and intercorrelations of the subscales).

### Therapists’ Treatment Experience with Minor-Attracted Persons

In terms of specific treatment experience with MAPs, 41.7% of the therapists indicated some experience with MAP patients (male therapists had more MAP treatment experience than female therapists; Table 4). Among those with MAP treatment experience, most therapists had treated male MAPs exclusively with only 6.3% of all therapists reporting having treated female MAPs. The median reported number of male MAPs therapists reported having treated was two, revealing that the majority of therapists had only limited treatment experience with this patient group. Therapists with MAP experience indicated that every fifth MAP patient had revealed their sexual interest in minors voluntarily right at the outset of the therapy with one fifth of treated MAPs admitting having used child sexual exploitation material and 5% acknowledging contact child sexual abuse. Notably, early admission of sexual interest in minors and acknowledging child sexual exploitation material use was more frequently reported by male than by female therapists (see Table 4 for further details on prior MAP treatment experience).

In case therapists were not willing to treat MAPs or could not accommodate the patients in their caseloads, the large majority (82.7%) indicated referring these patients to a different treatment institution. Out of the 74 therapists who indicated they did not redirect their MAP patients whom they could not treat, 68.9% agreed that they did not know to where they could refer the patients (14.9% were uncertain).

### Treatment Willingness, Treatment Competence, and Treatment Barriers

Outpatient therapists were markedly more willing to treat non-offending MAPs than MAPs who had committed any sexual offenses against children (contact or online offending); Wilcoxon signed-rank *W* = 191,280, *p* < .001, *r* = .94. For non-offending MAPs, roughly equal proportions of therapists were willing (47.6%) or unwilling (44.5%) to treat these patients. This shifted to 29.5% indicating willingness to treat as opposed to 62.7% indicating unwillingness to treat in case of patients who admitted past sexual offending against children. Strikingly, 39.3% of the therapists categorically ruled out any treatment willingness for offending MAPs by indicating a 1 on the Likert scale (as opposed to 21.3% who indicated a 1 in case of non-offending MAPs).

Outpatient therapists without treatment experience for MAPs indicated considerably lower general treatment competence for psychological problems associated with sexual interest in minors than their colleagues who had already treated MAPs; Mann–Whitney *U* = 10,065, *p* < .001, *r* = − .55. Strikingly, 87.5% of the therapists without MAP treatment experience indicated they did not have general MAP treatment skills as opposed to only a minority (7.6%) who reported having these skills. Nearly half of therapists (46.6%) who reported therapeutic experience with MAPs also disagreed with this item, indicating they perceived their general MAP treatment skills as lacking (as opposed to 24.3% agreement), attesting to the low levels of therapists’ perception of their own competence to treat MAPs. In line with this finding, only 15.2% of the therapists affirmed they had acquired any specific treatment skills for MAPs. Therapists who indicated having treatment experience with MAP patients reported higher levels of specific treatment skills than those who had treated MAPs; *χ*^2^(1) = 76.0, *p* < .001, *r*_ϕ_ = .42. Among therapists without MAP treatment experience, nearly all (97.6%) reported lacking specific treatment skills, but only about one-third of therapists who had worked with MAPs indicated lacking specific treatment skills. In terms of therapists’ reported sources of acquiring specific training for treating MAPs, supervision, studying the literature, training on the job, and specific training curricula were the top-ranked educative sources, whereas general therapist training or medical residency and therapists’ university studies were least frequently mentioned (all in descending order).

**Fig. 1:**
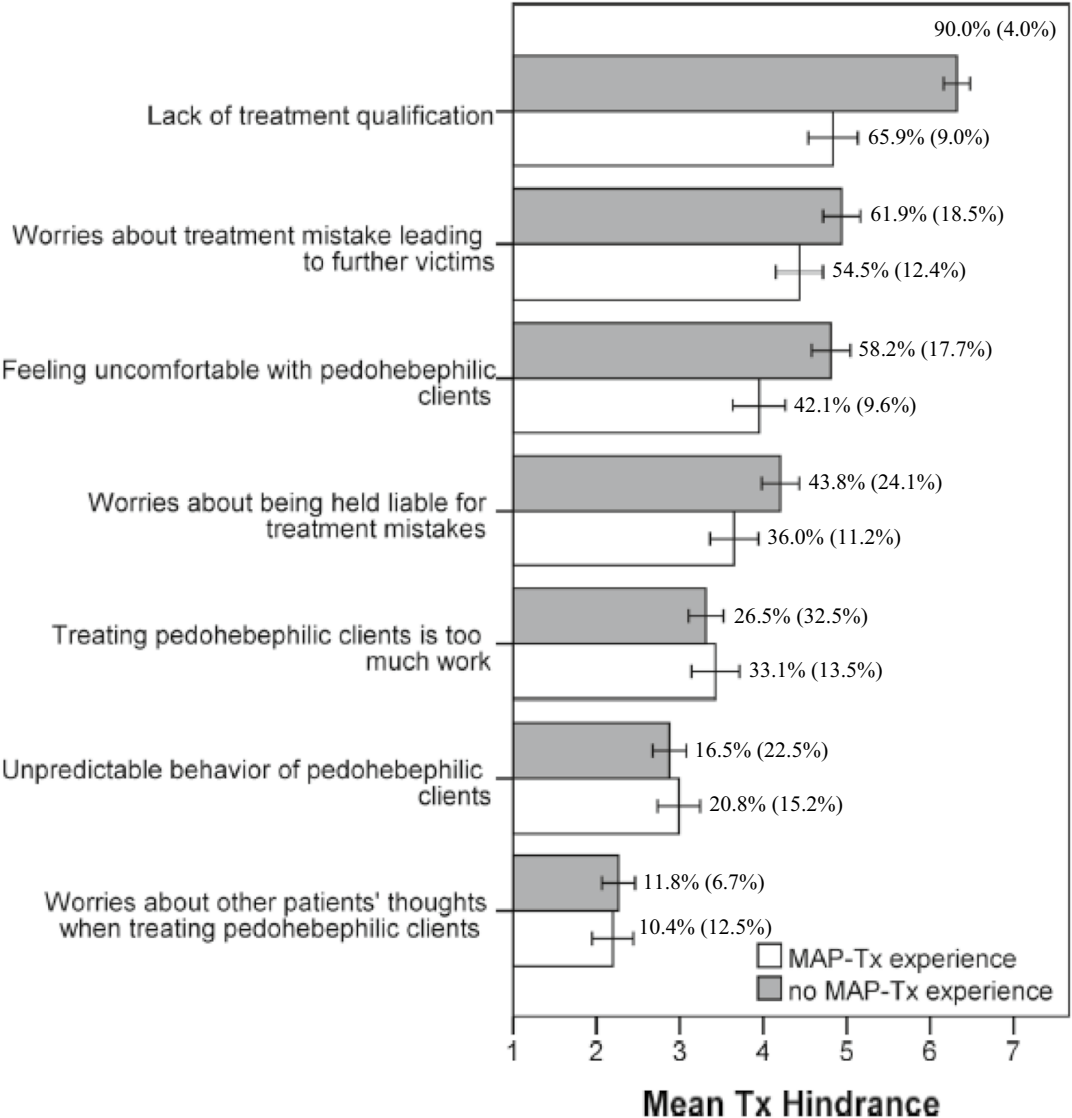
Overview of perceived treatment barriers as a function of MAP treatment experience (error bars ± CI_95%_) and percentages of agreement (and uncertain answers)

These perceived low MAP-specific treatment skills corresponded with the ranking of therapist-perceived treatment barriers (Fig. 1). Therapists regarded their lack of specific treatment qualifications as the most prevalent obstacle to treating MAPs followed by fear of treatment mistakes that might result in sexual victimization of children, feeling uncomfortable with MAPs as clientele, and worries about being held liable for treatment mistakes. All these worries were larger in participants who had no MAP treatment experience than in participants who already had treated MAPs (*p*s ≤ .007, *r*s ≥ .17). Therapists worried the least about what their other clients would think if they found out the therapist also treated MAPs. Relatedly, comparing the corresponding treatment barrier subscales, endorsement of Skills and Liability was substantially higher than endorsement of Effort, Wilcoxon signed-rank *W* = 84,386.5, *p* < .001, *r* = .92.

### Perceived Patients’ Problem Severity and Therapeutically Acceptable Sexual Behaviors

Ranking MAPs’ psychological problems by perceived severity, outpatient therapists regarded problems with intimacy as the most impaired symptom domain, followed by interpersonal, affective, and sexual and behavioral disorder symptoms (see Table 3 for the corresponding means and Fig. S1 in the electronical supplement for the full rank order of all items). In terms of possible sexual outlets according to Swiss legal standards, nearly half of the therapists (47.3%) saw MAPs’ masturbation to legal depictions of minors as problematic (vs. 29.3% who felt this was unproblematic). For legal child sex doll use, 51.5% rated this as problematic (vs. 17.9% who did not). Roughly two-thirds (68.1%) endorsed the usefulness of involving MAPs’ family members in the therapy process whereas only every tenth therapist (9.2%) rejected this.

### Stigmatizing Attitudes Toward Non-Offending Minor-Attracted Persons

Table 2 gives an overview of outpatient therapists’ stigmatizing attitudes toward non-offending MAPs in terms of agreement and uncertainty rates as well as available comparison data for the SDS and Intentionality scale from Jahnke et al., (2015a, 2015b, 2015c) and Koops et al. (2016). Descriptively, the large majority of surveyed therapists did not believe that MAPs have deliberatively chosen to develop their sexual interests (less than 5% agreed to any item measuring intentionality and over 85% disagreed with the related statements; Table 2). Comparisons with German community citizens and Russian sex therapists among whom at least 20% believed that pedophilic interests were intentionally chosen by MAPs revealed that significantly fewer Swiss therapists shared such beliefs (*p*s < .001, *d*s ≥ .80; Table 2).

Although, the large majority of Swiss outpatient therapists (75.4%) affirmed being willing to talk to non-offending MAPs (note that this means that still nearly a quarter were unsure or unwilling to do so), 40.5% indicated they would explicitly accept non-offending MAPs as a neighbor, 31.2% indicated they would accept the person as a colleague, and 27.6% indicated willingness to accept the person as a friend. Swiss therapists showed significantly less preference for social distancing from non-offending MAPs than German community citizens or Russian sex therapists (*p*s ≤ .002, *d*s ≥ .47, with the only exception of Russian sex therapists who did not differ from Swiss therapists in terms of their acceptance of MAPs as friends; Table 2). Notably, the rate of individuals agreeing to be willing to talk to non-offending MAPs was more than twice as high in Swiss mental health professionals than in both comparison groups. For the other SDS items, this difference was even more pronounced corroborating that although stigmatizing attitudes toward non-offending MAPs were present in Swiss therapists to some degree, these attitudes were noticeably attenuated compared to attitudes of German community citizens and Russian sex therapists (with the latter group revealing the most pronounced stigmatizing attitudes toward non-offending MAPs).

In terms of non-offending MAPs’ perceived dangerousness to children, the majority of Swiss therapists (58.8%) affirmed that a strong link exists between sexual interest in children and child sexual abuse and roughly one in five agreed that sexual interest in children will sooner or later lead to child sexual abuse (20.1%) or that many who have sexual interests in children will also have sex with children (19.4%; Table 2). Concerning punitive attitudes the large majority (84.7%) agreed that non-offending MAPs should not be allowed to work with children, and 40.3% believed that they should undergo mandatory psychotherapy. Roughly a quarter (26%) affirmed that citizens should be informed in case sexual offenders against children move into their neighborhood. However, only a minority opted for psychopharmacological “castration” (8.7%), preventive detention (6.8%), or openly accessible sexual offender registries (4.7%). Finally, aspects that related to deviancy were strongly affirmed by Swiss outpatient therapists: 80.3% believed that non-offending MAPs needed treatment, 57.1% agreed that these patients were sick, and 48.9% ruled out that they were normal with just rare sexual inclinations (Table 2).

### Zero-Order Intercorrelations

Table 3 displays the intercorrelations of all study variables. Taking into account that due to the large sample size even very small correlations were statistically significant, only at least conventionally small univariate correlations (Kendall’s *τ* ≥ .10) are interpreted here. All stigmatization-related variables were positively intercorrelated—with the exception of perceived deviance and intentionality of sexual interests. Stigmatizing attitudes were related to being a female therapist (with the exception of Intentionality and Dangerousness), decreased treatment willingness (except for Intentionality) and treatment competence (with the exception of Dangerousness, Intentionality, and Deviance). In terms of perceived treatment barriers, stigmatizing attitudes were associated with considering MAP treatment more effortful and (with the exception of Intentionality) greater therapist skill deficits and liability concerns. Moreover, stigmatization was linked to perceiving sexual and behavioral dysregulation among MAPs as well as increased interpersonal problems (the latter with the exception of Intentionality). However, this was not the case for affective and intimacy problems which were unrelated to stigmatizing attitudes except for a correlation between the Intimacy Problems and Deviance subscale.

Having any treatment experience with MAPs was correlated with being a male therapist, longer therapeutic treatment experience, fewer perceived treatment barriers due to skills and liability concerns, less punitiveness and social distancing, as well as increased treatment willingness and increased perception of interpersonal problems on the side of MAPs. The single strongest correlate of MAP treatment experience was perceived treatment competence (Kendall’s *τ* = .43). Self-reported treatment willingness was further associated with being a male or medical therapist, higher MAP treatment success expectations, and fewer perceived treatment barriers with, again, treatment competence being the strongest treatment willingness correlate (Kendall’s *τ* = .56). Perceived treatment barriers due to skills and liability concerns were linked with lacking treatment willingness and being a younger or female therapist. This pattern of intercorrelations suggests that particularly perceived MAP treatment competences and reduced stigmatizing attitudes—especially punitive attitudes, perceived deviance, and social distancing—as well as a lack of fear of liability concerns were among the most central indicators of positive attitudes toward therapeutically working with MAPs.

### Network Analysis

**Fig. 2:**
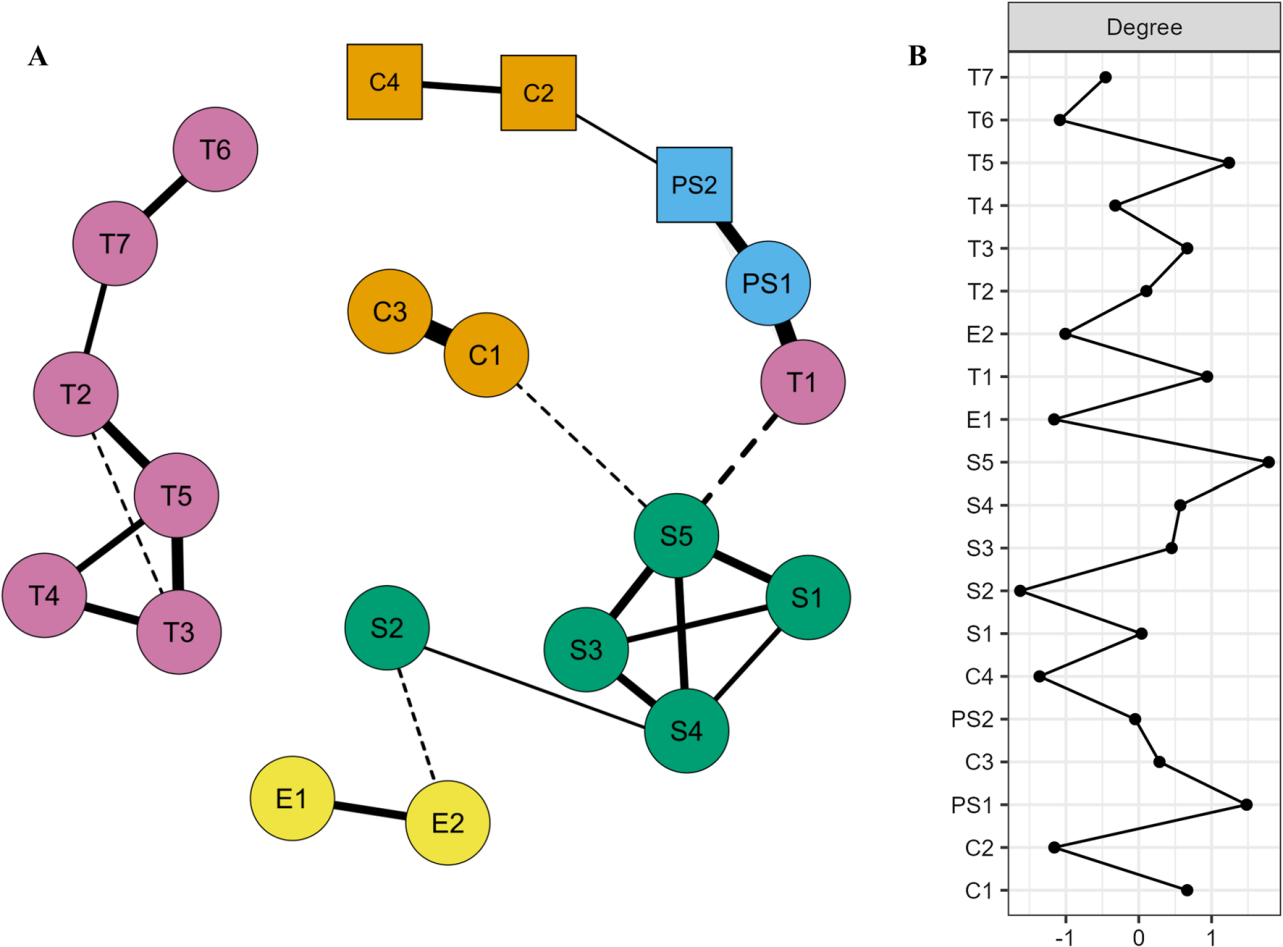
Network analysis plot (panel A) including z-standardized node centrality profile (panel B). *N* = 415 due to missing data. Quadratic nodes represent categorical variables. Edge thickness indicates strength of node association conditioned on all other possible associations between nodes (largest edge: C1–C3 = .56, smallest nonzero edge: S2–E2 = − .04). Negative associations are indicated by dashed lines; *control variables:* C1 = age, C2 = sex, C3 = general treatment experience (years), C4 = psychological vs. medical therapist; *stigmatization scales:* S1 = Dangerousness, S2 = Intentionality, S3 = Deviance, S4 = Punitive Attitudes, S5 = Social Distance Scale; *secondary prevention expectations:* E1 = success expectation, E2 = belief that more prevention is needed; *specific treatment skills:* PS1 = MAP treatment competence; PS2 = MAP treatment experience; *treatment expectations;* T1 = treatment willingness; T2 = MAP problem severity sexual and behavioral; T3 = MAP problem severity intimacy; T4 = MAP problem severity affective; T5 = MAP problem severity interpersonal; T6 = treatment barriers skills and liability; T7 = treatment barriers effort

Figure 2 displays the network of observed variables represented by nodes (circles, squares) that are connected by edges (lines) denoting strength of links between the nodes after statistically controlling for relationships with all other nodes, i.e., any remaining edge is adjusted for all other possible intercorrelations in the network. Out of 190 possible edges in a fully interconnected network of 20 nodes, 24 edges emerged as absolutely larger than zero (fraction of zero edges = .87) resulting in a relatively sparse network consisting of only few influential nodes.

Two main clusters of interrelated nodes were revealed. We detected a moderately intercorrelated *stigmatization cluster* that consisted of the stigma subscales Punitive Attitudes, Dangerousness, Deviance, and the SDS (lower right part in Panel A in Fig. 2). Perceived intentionality of sexual interest in children was indirectly linked to the stigmatization cluster through punitive attitudes. As expected, the stigmatization cluster was indirectly linked to MAP treatment willingness (slightly negatively) through the SDS. Moreover, this cluster was indirectly (slightly negatively) linked to therapist age (which, in turn was strongly related to therapist experience) also through the SDS. Only one other (weakly negative) edge emerged that indirectly connected the stigmatization cluster through intentionality of sexual interest in children with a set of two strongly positively related nodes tapping into beliefs of the effectiveness of MAP treatment (i.e., success expectations, belief that more secondary prevention is needed). Surprisingly, these latter two interrelated nodes exerted no further influence in the network.

In addition, a *MAP treatment-related expectations cluster* emerged (left part in Panel A of Fig. 2). This cluster consisted of therapists’ expectations concerning barriers faced in treatment when working with MAPs and the severity of typical problems exhibited by MAPs seeking treatment. In this cluster, MAPs’ interrelated intimacy, affective, and interpersonal problems were linked to therapists’ perceived treatment barriers through therapists’ perception of expected sexual and behavioral mental disorders among MAPs. Surprisingly, this cluster was not related to any further variable in the study which means that neither willingness to treat MAPs nor stigmatization of MAPs were linked to perceived treatment barriers or severity of MAPs perceived therapeutic problems.

Neither therapist sex nor profession (psychological vs. medical therapists) turned out to be related to MAP treatment-related attitudes (i.e., treatment willingness, perceived MAP problem severity, treatment barriers, expectations toward secondary prevention measures) or the main cluster of interrelated stigmatizing attitudes (i.e., perceived dangerousness, deviance, social distance as well as punitive attitudes, described below) from a multivariate perspective. In line with our hypotheses, willingness to therapeutically work with MAPs was most strongly linked to therapists’ perceived treatment competences for this clientele (which, in turn, was linked to having experience working with this group). Of note, controlling for multivariate relationships, being female was weakly related to past experience in working with MAPs (in opposition to what the univariate correlation revealed).

Finally, in terms of node centrality (strength) that is the absolute aggregation of all edge weights connected to a node (irrespective of their signs), the SDS, specific MAP treatment competences, and severity of MAPs’ perceived interpersonal problems emerged as the most influential network nodes (Panel B in Fig. 2). Strikingly, two of these central nodes were directly associated with treatment willingness, namely specific MAP treatment competences and SDS. Stability analyses based on case-dropping bootstrapped centrality (strength) estimates corroborated that the estimations were robust, with a centrality stability coefficient of .50, indicating that 50% of the data could be dropped to retain with 95% certainty a correlation of .75 with the original dataset. Moreover, edge weight estimations also turned out to be stable as the non-parametrically bootstrapped confidence intervals were rather narrow (largely ≤ .20 with only one notable exception for the C2–C4 edge being roughly twice as large).

## Discussion

Based on prior findings that MAPs report fears of and/or having experienced stigmatization from mental health professionals (e.g., B4U-ACT, 2011; Grady et al., 2019; Levenson & Grady, 2019a), we were interested in elucidating Swiss outpatient therapists’ actual experiences with and attitudes toward working with this clientele. Utilizing an anonymous online survey, we specifically sought to explore the multivariate interplay of stigmatizing attitudes toward MAPs with MAP treatment-related attitudes and experiences outside of Anglophone healthcare settings within a network analysis for the first time in the literature. Selecting for a relatively homogeneous sample of mental health experts delivering mental healthcare and treatment under less restrictive administrative conditions (i.e., with less strict mandatory reporting laws) who should be more open toward this highly stigmatized clientele due to their therapeutic identification and the more liberal regulatory framework, we were interested in exploring whether the same variables that have been studied in the prior literature would be associated with therapists’ willingness to treat MAPs.

### Therapist Stigmatization of Minor-Attracted Persons

First, we were interested in the actual amount of stigmatization of *non-offending* MAPs among mental health professionals in routine healthcare settings. Although Swiss therapists expectedly differed from German community citizens (Jahnke et al., 2015a) and from a small sample of highly stigmatizing Russian sex therapists (Koops et al., 2016) in terms of decreased beliefs that MAPs have deliberately chosen their sexual inclinations and concerning lesser preferred social distance toward MAPs, we found evidence for large individual differences in outpatient therapists’ reported stigmatization levels. Considerably large subgroups of therapists showed highly stigmatizing attitudes toward non-offending MAPs. Although roughly 60% of the respondents correctly acknowledged a link between sexual interest in minors and sexual victimization of children in community men (e.g., Klein et al., 2015), one fifth of the participants were convinced that non-offending MAPs’ sexual interests will *ultimately* lead to child sexual abuse and that MAPs will *eventually* have sex with children (Table 2). These knowledge deficits (see Dombert et al., 2016 and Joyal & Carpentier, 2021 for empirical concordance rates of sexual interest in children and child sexual abuse in community samples) corroborate the widespread conflation of sexual interest in minors and sexual victimization of children even among a subgroup of mental health professionals in less restrictive treatment contexts (Jahnke, 2018a; Lievesley et al., 2022). Although non-offending MAPs were largely regarded as individuals who had not intentionally chosen their sexual interests, non-negligible groups of therapists shared public beliefs (although at a statistically lower rate than the general public) that MAPs are dangerous, deviant (i.e., the majority believed MAPs are sick and in need of treatment), and need to be controlled by punitive administrative measures such as informing citizens about child sexual offenders becoming their neighbors (26%) or subjecting them to mandatory psychotherapy (40%).

Notably, in univariate analyses female therapists emerged as reporting higher MAP deviancy, increased punitiveness against MAPs, and stronger social distancing from MAPs than their male colleagues while at the same time indicating that the mostly male MAPs treated by them were initially less revealing of their sexual interests in minors and their child sexual exploitation material use. The preliminary finding concerning male MAPs’ reduced openness with female therapists should be followed up in future research in order to see whether it is indeed a robust finding and not due to some confound by third variables associated with therapist sex in our study (as suggested by the lack of any respective link to therapist sex in the network analysis).

### Treatment Willingness, Treatment Competence, and Problem Severity

Although roughly 42% of the surveyed outpatient therapists reported having treated at least one MAP patient, their treatment experience with this clientele was still limited (i.e., the median number of treated MAPs among therapists who had treatment experience with this clientele was two). Therapists strongly believed in the beneficial effects of secondary prevention programs for MAPs (*M* > 6 on a seven-point scale; Table 4) and the large majority were willing to refer patients to different treatment institutions which corresponded with therapists’ strong beliefs in non-offending MAPs’ need for therapeutic treatment. In spite of therapists’ very positive notions about the effectiveness of secondary prevention for MAPs, they were roughly equally split when it came to their personal willingness to take on non-offending MAPs for treatment. As hypothesized, this proportion became significantly more unbalanced with roughly twice as many unwilling than willing therapists in case of MAPs who acknowledged past sexual victimization of minors. Notably, roughly 20% versus 40% of Swiss outpatient therapists categorically ruled out any treatment willingness for non-offending and offending MAP patients, respectively.

Adding evidence to recent findings from more heterogeneous Anglophone samples of health professionals (Lievesley et al., 2022; Roche et al., 2022), we were furthermore able to corroborate our hypotheses that psychiatric and psychological mental health professionals’ stigmatizing attitudes toward MAPs and their perceived lack of specific treatment competencies for MAPs are associated with their reduced willingness to treat MAPs (Fig. 2). This corresponds to our finding that roughly twice as many therapists without vs. with MAP treatment experience felt incompetent for treating this specific clientele, but only a minority felt clearly competent (7% without vs. 24% with MAP treatment experience) to do so. Similarly, almost all therapists who had never treated MAPs indicated they did not have any specific training for this patient group (as opposed to 33% among those with MAP treatment experience). Perceived lack of MAP-specific treatment competence was regarded as the topmost treatment obstacle for taking on MAPs as patients and treatment obstacles were indicated as being larger among therapists without vs. with treatment experience (Fig. 1). These findings underscore the importance of the pervasively perceived lack of MAP-specific knowledge (Lievesley et al., 2022; Roche & Stephens, 2022) and treatment skills for dealing with sexual attraction in minors among outpatient routine care mental health professionals.

Noteworthy, although Swiss mental health professionals identified sexual and intimacy needs as well as fear of stigmatization as MAPs’ most severe psychological problems (the latter finding becomes noteworthy given the amount of MAP stigmatization reported by the therapists themselves), other typical problems that are of primary concern to MAPs from their own perspective were ranked as less important (i.e., loneliness, anxiety, depression, lack of life satisfaction, problems with general and sexual self-regulation apart from sexual offending; Levenson et al, 2020). This exemplifies the–at least partly–diverging therapeutic foci between therapists and patients as therapists seem to prioritize sexual behavior regulation issues and sexual offense prevention, whereas MAPs experience problems with psychological well-being and stigmatization issues as more crucial (B4U-ACT, 2011; Levenson & Grady, 2019a). This divergence in therapeutic foci is likely among the reasons reason why MAPs are reluctant to seek professional help or report dissatisfaction with it (Levenson & Grady, 2019a). This is an important aspect that needs to be considered by outpatient therapists because patient-therapist goal consensus and collaboration are important indicators of a functioning therapeutic alliance that facilitates positive treatment outcomes and prevents patient dropout (Tryon et al., 2018).

### Interplay of Stigmatization, Treatment Willingness, and Therapist Characteristics

Because most stigma variables and therapy-related attitudes and experiences were theoretically meaningfully intercorrelated (Table 3), we explored the multivariate interplay utilizing network analysis. Once multivariate intercorrelations with all other variables in the network were controlled for, the present data yielded a relatively sparse network with only few statistically relevant associations consisting of two major clusters of interrelated variables: Treatment-related expectations and stigmatizing attitudes (Fig. 2).

Importantly, therapist sex and profession (psychological vs. medical therapists) as well as therapists’ overwhelmingly positive attitudes toward secondary prevention were not related to either of the two clusters. The stigmatizing attitudes cluster consisting of the interrelated SDS and Deviance, Punitive Attitudes, and Dangerousness stigma subscales was negatively related to therapist age and general treatment experience. The most relevant findings, however, should be considered that—as hypothesized—(a) stigmatizing attitudes were linked to reduced treatment willingness and (b) perceived MAP treatment competence was by far the largest direct correlate of increased treatment willingness. Interestingly, perceived treatment barriers and MAPs’ perceived problem severity yielded no relationship with treatment willingness or with stigmatizing attitudes.

### Implications for Increasing Therapists’ Treatment Willingness

Due to the cross-sectional study design, we cannot draw inferences about the causality of the emerging network links but the data may suggest that the prior focus on anti-stigma interventions in the literature (e.g., Jahnke et al., 2015b; Levenson & Grady, 2019b; Walker et al., 2022) will likely be only partly successful in increasing therapists’ willingness to treat MAPs. This is corroborated by the relatively weak direct link from the SDS to treatment willingness as opposed to the much stronger link to subjectively perceived specific MAP treatment skills (Fig. 2). To this end, providing therapists with information and training that enhances their perceived MAP-specific knowledge and treatment competence (e.g., Jahnke, 2018a; Levenson & Grady, 2019a; Levenson et al., 2020) should be considered at least equally important if professional help offers for MAPs shall become more widely available among routine mental healthcare providers. This is in line with the findings that the topmost affirmed treatment barrier was lack of specific treatment skills and perceived MAP treatment competence was the single strongest correlate of willingness to treat MAPs.

Notably, it is surprising that treatment-related expectations concerning barriers and severity of differential psychological problem areas were unrelated to treatment willingness and stigmatizing attitudes as well as any therapist characteristics in the network analysis. This non-relatedness of therapists’ concepts of the problems exhibited by MAPs and the reasons why it should be difficult to treat them resonates with one anonymous reviewer’s doubts about whether outpatient therapists indeed do need specific training for treating (primarily non-offending) MAPs. It is an open empirical question how much specific training is in fact necessary for effective psychotherapy with MAPs who voluntarily seek help. It is important to point out, however, that outpatient therapists (un)willingness to work with this group will likely be driven by their *subjectively perceived* expertise (and not so much by their actual level of unspecific therapist training that should practically suffice in dealing with most MAPs’ psychotherapeutic needs in the narrow sense). This might indicate that there is a strong belief among non-specialized mental health practitioners that MAPs are psychologically fundamentally different from their regular patients due to the specifics of their sexual inclinations. In fact, prevalent psychological problems in routine care patients such as decreased life satisfaction, depression, anxiety, substance abuse, or loneliness resemble the problems experienced by many MAPs (Jahnke, 2018a; Lawrence & Willis, 2021). Relatedly, MAPs seek quite the same qualities in their psychotherapists as routine care patients in order to profit from their treatment (Levenson & Grady, 2019a).

Nevertheless, we believe that at least in terms of basic sexology, psychopathology, epidemiology, and the empirical nature of the link between pedohebephilic interest and sexual offending against children, the present data underscore that there is headroom for advancing outpatient therapists’ MAP-related knowledge. Accordingly, worries about treatment mistakes leading to further sexual victimization of minors, feeling uncomfortable with this clientele, and worries about being held liable for treatment mistakes were among the most relevant treatment barriers that have been indicated by our participants. Specifically, worries and doubts about how to deal with MAPs who are perceived as (imminently) dangerous underscore widespread knowledge deficits among therapists concerning ethical and legal boundary conditions, especially in terms of reporting duties and possibilities in their respective legal frameworks (Beggs Christofferson, 2019; McPhail et al., 2021; Stephens et al., 2021; Walker et al., 2022). Thus, outpatient therapists (and their openness toward MAPs as clients) might profit from shifting their therapeutic perspective. Rather than pondering the yet dominating question in MAP treatment of *whether* someone with pedohebephilic sexual interests will victimize children, therapists should focus on the question *under which specific boundary conditions* their clients might (or, importantly, might not) pose a risk to children and *how these specific dynamic risk factors can be therapeutically dealt with*–if necessary at all in an individual case. Given the fact that child sexual abuse is prevalently committed also by non-pedohebephilic individuals (e.g., Schmidt et al., 2013), this implies adequate knowledge about relevant risk factors. Such basic criminal psychological facts, however, are not part of current general clinical training curriculae in psychology or medicine and should help to keep the prevailing risk focus in check. This may be conducive to setting the stage for recognizing other concerns that lead MAPs to seek therapeutic help.

### Limitations and Outlook

It has to be noted that due to self-selection processes in participation with the online survey, the study sample is not representative of Swiss outpatient therapists working in routine care. Specifically, with 42% of the sample reporting at least limited experience working with MAPs it seems likely that there was an overrepresentation of therapists who already had worked with this clientele (and, thus, have at least been willing to take up these patients for treatment). Hence, we believe, that our findings in terms of the reported non-willingness and stigmatization levels are rather conservative estimations of the actual stigmatizing attitudes toward MAPs particularly among therapists who have never treated MAPs (which should consist of the large majority of eligible therapists given the low prevalence of pedophilic sexual inclinations in the population; Dombert et al., 2016). Moreover, due to the relatively small sample from the perspective of network analyses (Borsboom et al., 2021) results from this particular analysis have to be considered as tentative and call for further replication (but note that our bootstrapped stability analyses supported the robustness of the reported results). Furthermore, it has to be noted that three scales (Dangerousness, Deviance, Belief that More Secondary Prevention is Needed) showed internal consistencies below .60 and thus need to interpreted cautiously. We attribute this finding to the mix of therapists varying in levels of expertise and experience with MAPs who have answered these items in an inconsistent manner (e.g., more experienced therapists likely will endorse that there is a statistical link between sexual interest in children and child sexual abuse in community members but will not believe that this necessarily leads to sexual victimization of children in any case). Nevertheless, our sample is by far the largest and most homogeneous sample of mental health experts that has hitherto been reported in research on therapist stigmatization of MAPs.

In summary, our results further corroborate that MAPs’ reported fears of being stigmatized by (mental) health professionals are not unfounded (B4U-ACT, 2011; Levenson & Grady, 2019a). Based on our sample of exclusively mental health professionals, it now becomes clear that the reluctance to treat MAPs is indeed linked to the specific stigmatization of this clientele and not just a function of general stigmatization of mental health patients that might drive general healthcare practitioners’ rejection of psychotherapy clients. Paradoxically, although routine care therapists strongly believe in the utility of psychotherapy for the prevention of child sexual abuse and that MAPs indeed do need therapeutic assistance, a considerable subgroup of therapists harbors strong reservations against personally providing MAPs with such professional help. In order to increase the chances of this underserved clientele finding access to professional help to deal with their considerable burden of psychological distress (Lawrence & Willis, 2021), particularly outpatient therapists who yet refuse to work with MAPs as well as future therapists in training should be made aware of the principles outlined in B4U-ACT (2020), Jahnke (2018a), and Levenson et al. (2020) who recommend guidelines to decrease stigmatization of MAPs and increase professional certainty and competences in therapeutically working with them. To this end, one important step forward would be to include MAPs into the development of treatment opportunities directed at them and of trainings for therapists as well (Stephens et al., 2020).

## Supplementary Information

Below is the link to the electronic supplementary material.

Supplementary file1 (DOCX 77 KB)

## Data Availability

The survey and item material are available upon request from the researchers.

## Footnotes

1 The age of legal consent for sexual activity in Switzerland is 16 years.

2 This should not be confused with the well-established finding that pedophilic interest is one of the best prospectively validated risk factors *for sexual reoffending among people who have already been convicted of sexual offences* (Mann et al., 2010).

3 The Swiss legal framework (similar to Germany) guarantees far-reaching confidentiality for therapy patients. Only in case of imminent danger for a concretely identifiable victim of child sexual abuse are therapists allowed to breach confidentiality. Past child sexual abuse and (even current) child sexual exploitation material use does not suffice to breach this patient right unless specific victims can be identified.
