# Supplementary material for: Outpatient Therapists’ Perspectives on Working With Persons Who Are Sexually Interested in Minors
Source: Arch Sex Behav. 2022 Aug 8;51(8):4157–78. doi: 10.1007/s10508-022-02377-6 (PMC9663344; doi:10.1007/s10508-022-02377-6)

**Electronical Supplement**

| **Table S1**  Factor loadings (pattern matrix) for perceived severity of minor-attracted persons’ treatment-relevant probems | | | | | | | | | | | |
| --- | --- | --- | --- | --- | --- | --- | --- | --- | --- | --- | --- |
|  | | **Factor 1** | | **Factor 2** | | **Factor 3** | | **Factor 4** | | **Uniqueness** | |
| Hypersexuality |  | 0.77 |  |  |  |  |  |  |  | 0.43 |  |
| Sexual dysfunctions |  | 0.70 |  |  |  |  |  |  |  | 0.52 |  |
| Chronic antisociality |  | 0.68 |  |  |  |  |  |  |  | 0.45 |  |
| Psychotic spectrum |  | 0.66 |  |  |  |  |  |  |  | 0.44 |  |
| Cognitive disabilities |  | 0.63 |  |  |  |  |  |  |  | 0.60 |  |
| Depression |  |  |  | 0.94 |  |  |  |  |  | 0.19 |  |
| Social anxiety |  |  |  | 0.81 |  |  |  |  |  | 0.34 |  |
| Loneliness |  |  |  | 0.60 |  |  |  |  |  | 0.52 |  |
| Substance abuse |  |  |  | 0.49 |  |  |  |  |  | 0.57 |  |
| Intimacy with adults |  |  |  |  |  | 0.73 |  |  |  | 0.49 |  |
| Lack of legal satisfying sexual outlets |  |  |  |  |  | 0.71 |  |  |  | 0.55 |  |
| Keeping sexual interest in minors secret |  |  |  |  |  | 0.48 |  |  |  | 0.66 |  |
| Desisting child sexual exploitation material use |  |  |  |  |  | 0.35 |  |  |  | 0.79 |  |
| Personality disorders |  |  |  |  |  |  |  | 0.83 |  | 0.38 |  |
| Self-control/Emotion regulation |  |  |  |  |  |  |  | 0.68 |  | 0.47 |  |
| Prior child sexual abuse in childhood/youth |  |  |  |  |  |  |  | 0.44 |  | 0.68 |  |
|  | | | | | | | | | | | |
| *Note.*  Applied rotation method is promax. Factor loadings below .30 are suppressed for ease of interpretation. | | | | | | | | | | | |

| **Table S2**  Factor loadings (pattern matrix) for perceived MAP treatment barriers | | | | | | | |
| --- | --- | --- | --- | --- | --- | --- | --- |
|  | | **Factor 1** | | **Factor 2** | | **Uniqueness** | |
| Treatment errors resulting in victimization of children |  | 0.83 |  |  |  | 0.34 |  |
| Being held liable for treatment errors |  | 0.75 |  |  |  | 0.41 |  |
| Not being qualified enough to treat MAPs |  | 0.49 |  |  |  | 0.80 |  |
| Feeling uncomfortable working with MAPs |  | 0.45 |  |  |  | 0.65 |  |
| MAPs are unpredictable |  |  |  | 0.86 |  | 0.33 |  |
| MAPs are too labour-intensive |  |  |  | 0.69 |  | 0.57 |  |
| Worrying about other patient’s thoughts when becoming aware that MAPs being treated by therapist |  |  |  | 0.41 |  | 0.77 |  |
|  | | | | | | | |
| *Note.*  Applied rotation method is promax. Factor loadings below .30 are suppressed for ease of interpretation. | | | | | | | |

**Figures**

**Figure S1**

Overview of MAPs’ therapeutic problems ordered by perceived severity (error bars ± CI_95%_).


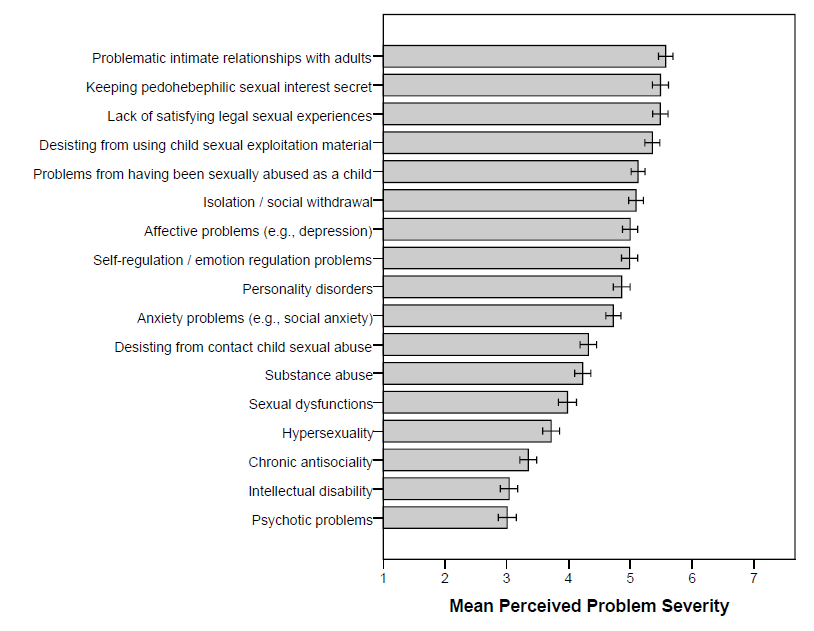

Supplement: Supplementary file 1 — Supplementary file1 (DOCX 77 KB) [file 10508_2022_2377_MOESM1_ESM.docx]
